# A Rare Case of Massive Gastrointestinal Hemorrhage Caused by an Ileal Gastrointestinal Stromal Tumor

**DOI:** 10.7759/cureus.82094

**Published:** 2025-04-11

**Authors:** Adarsh Jha, Amey Joshi, Divij K Jha, Ryan K Mui, Richa Tikaria, Tadd K Hiatt

**Affiliations:** 1 Internal Medicine, Michigan State University, East Lansing, USA; 2 Gastroenterology, Michigan State University, East Lansing, USA; 3 Gastroenterology, University of Michigan, Lansing, USA

**Keywords:** bleeding gist, gist, ileal gist, kit mutation, management of lower gi bleeding

## Abstract

Gastrointestinal stromal tumors (GISTs) are rare mesenchymal tumors of the gastrointestinal tract. Although gastrointestinal bleeding is a common complication of GISTs, life-threatening hemorrhage is rare, particularly with intestinal GISTs. We report a rare case of an ileal GIST in a 71-year-old female patient who presented with massive lower gastrointestinal bleeding. Initial CT angiography (CTA) demonstrated a brisk gastrointestinal bleed in the ileal region. Despite embolization of the associated distal ileal artery branches, the patient experienced persistent hemoglobin drops and hematochezia. Repeat CTA revealed a hyper-enhancing exophytic lesion in the proximal ileum. This 4.7cm ileal tumor had to be excised, and histopathology confirmed a low-grade GIST. This case highlights the diagnostic complexity of ileal GISTs and the importance of imaging modalities such as CTA in managing active gastrointestinal bleeding.

## Introduction

Gastrointestinal stromal tumors (GISTs) are rare neoplasms arising from the mesenchymal tissue of the gastrointestinal (GI) tract, accounting for approximately one to two percent of all GI tumors. They are most commonly found in the stomach and small intestine but can occur anywhere along the GI tract. GISTs often present with nonspecific symptoms, such as GI bleeding, abdominal pain, weight loss, or a palpable mass, and may also be discovered incidentally during imaging or surgical procedures [1]. Among these, GI bleeding is a significant clinical manifestation, particularly in cases involving vascular tumors.

GISTs are believed to originate from interstitial cells of Cajal or their precursors, which regulate gut motility. Most GISTs (about 85%) are characterized by mutations in the* KIT *(*CD117*)or platelet-derived growth factor receptor alpha (*PDGFRA*) genes, leading to activation of tyrosine kinase receptors and uncontrolled cell proliferation. A minority, referred to as "wild-type" GISTs, lack these mutations and may instead harbor alterations in genes such as *NF1, BRAF, *or* HRAS*. While the majority of GISTs are benign, malignant cases exhibit hematogenous spread, most commonly to the liver and peritoneum, whereas lymphatic metastasis is rare [2].

Imaging modalities such as CT angiography (CTA) are instrumental in identifying the source of bleeding in hemorrhagic presentations, as they can detect abnormal vascular patterns or contrast extravasation. Alternative imaging techniques, including 99mTc-labeled RBC scans and magnetic resonance angiography, may be used when CTA is inconclusive [3].

In this report, we present a rare case of an ileal GIST that manifested as massive lower GI bleeding, necessitating multiple blood transfusions. This case underscores the importance of early recognition, accurate imaging, and immunohistochemical diagnosis in managing GISTs, particularly when they present with life-threatening complications. 

This article was previously presented as a poster at the 2024 American College of Gastroenterology on October 25, 2024.

## Case presentation

**Figure 1:**
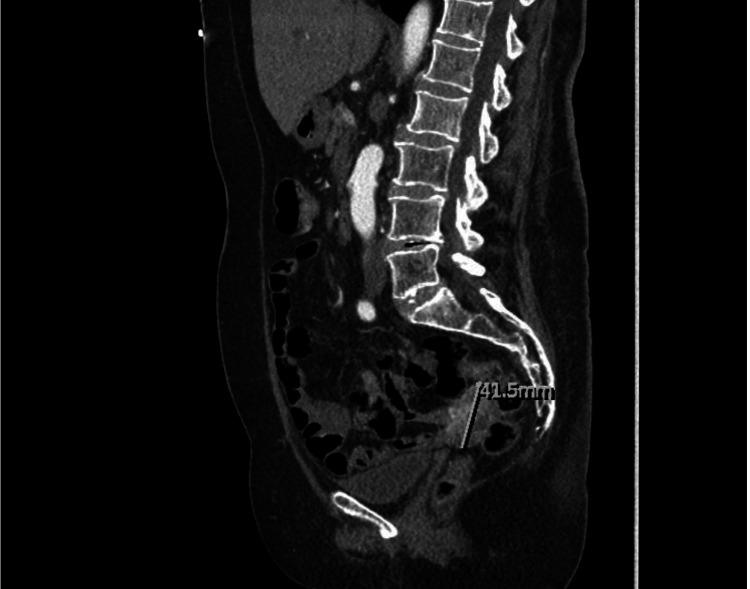
CT angiogram showing a heterogeneously arterial phase hyper-enhancing exophytic circumscribed lesion involving the ileum, measuring 4.7 x 3 x 4.1 cm

**Figure 2:**
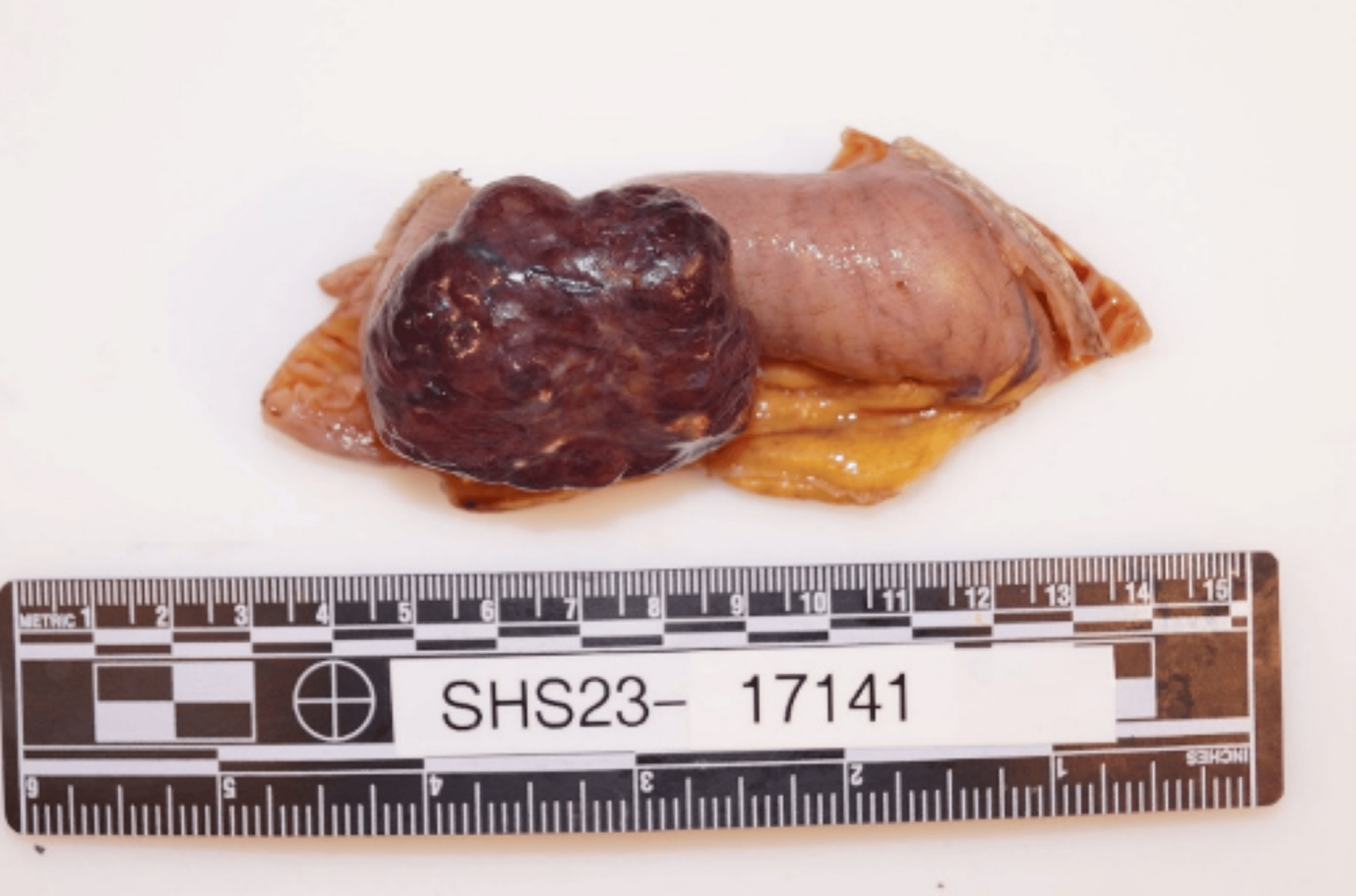
Gastrointestinal stromal tumor measuring 4.7 x 4.5 x 2 cm, with attached small bowel

**Figure 3:**
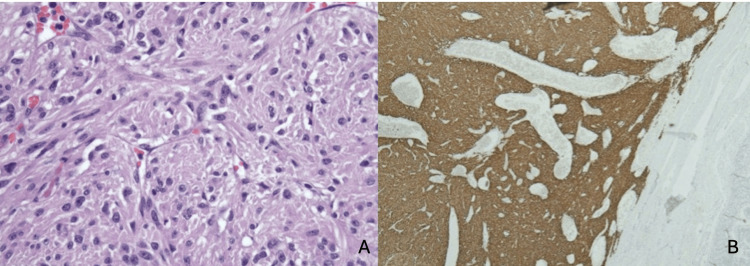
Histopathology images (A) H&E stain of the mass showing proliferation of spindle cells and (B) demonstration of strong diffuse staining with CD117 and DOG1

A 71-year-old woman with a past medical history of diverticulosis presented with complaints of multiple episodes of bright red bowel movements and diffuse abdominal pain for two days. On admission, the patient was afebrile (temperature 98.6°F) with a pulse rate of 101 beats per minute, respiratory rate 16 breaths per minute, blood pressure of 115/67 mmHg, and oxygen saturation of 97% on room air. She was noted to be anemic, with hemoglobin of 5.9 g/dl (normal range: 12-15 g/dl) and elevated BUN of 35 mg/dl (normal range: 6-23 mg/dl). CTA revealed a brisk gastrointestinal bleed in the ileal region (Figure 1). An emergent selective superior mesenteric angiogram was performed with embolization of the distal branches of the dominant ileal artery. However, the patient continued to have a persistent drop in hemoglobin levels and reported episodes of frank blood with clots in her stools. Subsequently, esophagogastroduodenoscopy was performed for further evaluation, but no active bleeding in the duodenum was revealed. A repeat CTA of the abdomen revealed a hyper-enhancing exophytic 4.7 x 3 x 4.1 cm lesion in the proximal ileal region. The patient subsequently underwent a laparoscopic excision of the mass with small bowel resection and primary anastomosis (Figure 2). The pathology sample confirmed a low-grade GIST with negative margins and positive immunohistochemical staining with *CD117* and *DOG1* (Figure 3). The patient had an uneventful postoperative course, and hemoglobin levels remained stable. On follow-up after 30 days, her hemoglobin levels remained stable, with no further reports of melena or hematochezia.

## Discussion

GISTs are one of the most common types of mesenchymal tumors of the GI tract, with an estimated incidence of 1.2 per 100,000 individuals [4]. Although GI bleeding is the most common complication associated with these tumors, occurring in around 30-40%, only a small percentage of these occur with life-threatening hemorrhage [4]. Gastric GISTs are more likely to cause gastrointestinal bleeding than intestinal GISTs [5]. The present case highlights this rare occurrence and complication.

GISTs are well-defined and exophytic tumors and comprise a pseudocapsule. Microscopically, GISTs can have a spindle cell or epithelioid morphology or mixed features. The immunohistochemistry of GISTs, a significant diagnostic measure, is positive for *KIT* (*CD117*) and *DOG1*. Molecular profiling commonly shows KIT exon 11 mutation with relatively uncommon profiling like *NF1* mutations and *SDH* deficiency. The uncommon profiling is associated with a poorer prognosis [6]. Our patient's pathology sample confirmed a low-grade GIST with negative margins and positive immunohistochemical staining for CD117 and DOG1. The tumor had extensive vascularity, and one of the larger vessels was thrombosed. This architecture is likely to have been the precursor for hemorrhagic presentation.

Imaging studies, primarily with CT imaging, play a pivotal role in diagnosing ileal GISTs. In cases of active gastrointestinal bleeding, CTA can help localize the bleeding by visualizing abnormal vascular patterns or contrast material extravasation. This diagnostic modality was beneficial in our patient with a brisk gastrointestinal bleed. Nuclear medicine scans like 99mTc-labeled red blood cell scans and magnetic resonance angiography can be helpful in cases where CTA is non-diagnostic [1,4,7,8]. The mainstay treatment for non-metastatic GISTs is resection. Minimally invasive techniques, including laparoscopic resection, are shown to be effective for gastric GISTs; however, long-term data are limited. Tyrosine kinase inhibitors like imatinib are the first-line therapy for unresectable, metastatic, or recurrent tumors, increasing survival rates. Adjuvant imatinib for three years is recommended for high-risk cases post-surgery, with advanced cases requiring a second-line tyrosine kinase inhibitor (sunitinib) or third-line multikinase inhibitor (regorafenib) [8].

Joensuu’s risk stratification system helps to determine high-risk tumors that likely benefit from adjuvant imatinib therapy, which is also the cornerstone for unresectable GISTs [9]. Studies such as the ASCOG 79001 and SSGXVIII/AIO trials have validated the efficacy of extended adjuvant therapy in reducing recurrence rates. The ASCOG Z9001 trial involving 713 patients with tumors over three cm showed recurrence-free survival rates of 83-98% at one year with adjuvant imatinib after resection of primary GI stromal tumor. However, overall survival remained similar between groups receiving adjuvant and non-adjuvant therapy. Additionally, the European SSGXVIII/AIO trial demonstrated that three years of adjuvant imatinib therapy resulted in superior outcomes compared to one year, with a five-year recurrence-free survival of 66% versus 48% and overall survival of 92% versus 82% [10].

Management of asymptomatic GISTs through minimally invasive procedures such as endoscopic enucleation is being studied; however, the risk of pseudocapsule injury, peritoneal seeding, and suboptimal resection exists. Techniques such as laparoscopy and endoscopy cooperative surgery and laparoscopy-assisted full-thickness resection contribute to reduced risks and a higher complete resection rate, making them safer than endoscopic procedures alone. Further study is required concerning minimally invasive procedures [11]. 

Mutations in *DOG1*, *KIT*, and *PDGFRA* have been associated with the prognosis of GISTs. Overexpression of *DOG1* has been linked to a proliferative advantage in malignant stromal cells. The presence of a homozygous *KIT* exon 11 mutation is also associated with a more aggressive disease course. This is supported by a recent meta-analysis of 1,487 patients, which found that GISTs with *KIT* mutations have a significantly poorer prognosis compared to those with *PDGFRA* mutations or wild-type GISTs. In contrast, the majority of *PDGFRA*-mutated GISTs are associated with a more benign clinical course. Another observational study reported a more favorable two-year post-operative recurrence-free survival rate in *DOG1*-negative patients compared to *DOG1*-positive ones. In that study, patients with strong *DOG1* expression, tumor size ≥ 5 cm, and mutations in *KIT* or *PDGFRA* were found to have a worse prognosis [12].

The prognosis of ileal GISTs is guided by the mitotic index, size, and rupture status. A large tumor with a high mitotic index poses a worse outcome. In high-risk cases, post resection, surveillance every three to six months is recommended for at least five years. Follow-up imaging is necessary to ascertain early detection in case of recurrence [13].

## Conclusions

Ileal GISTs carry significant challenges in terms of diagnosis, given their location, unlike gastric or esophageal GISTs. Early detection is pivotal for an increase in treatment success rates and survival. A multidisciplinary approach with investigations and management, including imaging, molecular characterization, and targeted therapies, has significantly improved patient outcomes.
